# Supplementary material for: Creating simple predictive models in ecology, conservation and environmental policy based on Bayesian belief networks
Source: PLoS One. 2024 Dec 10;19(12):e0305882. doi: 10.1371/journal.pone.0305882 (PMC11630608; doi:10.1371/journal.pone.0305882)
Supplement: Attachment — Submitted filename: Response to Reviewer_VDA.docx [file pone.0305882.s006.docx]

*We have left the reviewer’s comments in plain black font, our replies are in italics, and any quotes from the revised text of the paper are presented in red.*

Reviewer #1: Overall: The authors efforts in conducting the research and writing the manuscript are appreciated. However, the paper needs significant reorganization to ensure consistency with its aims and content, as well as a stronger methodology section. I believe developing a new version of the paper would be a better option.

The paper lacks a strong structure. Specifically, it is missing an abstract that summarises the objectives, methods, results, and conclusions. While the paper includes an introduction, it does not provide adequate background information and context, state the research problem or question, explain the significance and objectives of the study. Furthermore, it is highly recommended to include a literature review section that examines relevant previous research, identifies gaps in the existing literature, and demonstrates how the current research builds upon or differs from past studies. Additionally, the paper lacks both methodology and results sections, making it difficult for readers to fully understand the research.

*We thank the reviewer for their thorough reading of the paper. The paper presents work based on a new R analysis package, and explains the underlying theory of the package and examples for how it is used. PLoS ONE was chosen to submit to, as such studies are included in the journal’s scope. However, there are no formal results of this study, so it isn’t possible to structure in a traditional manner. We have made a number of changes to the paper based on the reviewer’s suggestions, which are detailed in later sections.*

Abstract: The abstract should clearly state the primary objective and contribution of the work, highlighting the significance of the new R package (BBNet) earlier on. It should better articulate how the proposed approach simplifies traditional models and clarify what is meant by the models not being fully quantitative. Additionally, the abstract should briefly describe the parameterisation process, mention specific examples of the model's application across different fields, and suggest potential future research directions or improvements.

*We thank the reviewer for these suggestions. The Abstract now reads:*

Predictive models are often complex to produce and interpret, yet can offer valuable insights for management, conservation and policy-making. Here we introduce a new modelling tool (the R package ‘*BBNet’*), which is simple to use, and requires little mathematical or computer programming background. By using straightforward concepts to describe interactions between model components, predictive models can be effectively constructed using basic spreadsheet tools and loaded into the R package. These models can be analysed, visualised, and sensitivity tested to assess how information flows through the system’s components and provide predictions for future outcomes of the systems. This paper provides a theoretical background to the models, which are modified Bayesian belief networks (BBNs), and an overview of how the package can be used. The models are not fully quantitative, but outcomes between different modelled scenarios can be considered ordinally (i.e. ranked from ‘best’ to ‘worse’). Parameterisation of models can also be through data, literature, expert opinion, questionnaires and/or surveys of opinion, which are expressed as a simple ‘weak’ to ‘very strong’ or 1-4 integer value for interactions between model components. While we have focussed on the use of the models in environmental and ecological problems (including with links to management and social outcomes), their application does not need to be restricted to these disciplines, and use in financial systems, molecular biology, political sciences and many other disciplines are possible.

Introduction: The introduction is packed with numerous points that make it hard to follow. It lacks a clear, concise narrative, making it overwhelming for readers to understand the amin focus. Also, it jumps between various concepts without clear transitions. However, consider breaking the introduction into more distinct paragraphs, each focusing on a specific point. For example, you could have separate paragraphs for:

• The current state of quantitative skills in the environmental workforce.
• The challenges of using sophisticated models and AI in ecology.
• The introduction and benefits of Bayesian belief networks (BBNs).
• The limitations of traditional BBNs.
• Recent advancements in BBNs and their applications.

*We have modified the introduction to the following paragraphs*

- *The mismatch between the lack of quantitative experience in most environmental scientists and policy makers and the complexity of building and interpreting models (i.e. a problem statement, which our work will address)*
- *The needs of policy makers and conservationists from predictive models*
- *The introduction and benefits of Bayesian belief networks (BBNs)*
- *The limitations of traditional BBNs.*
- *Recent advancements in BBNs and their applications.*
- *Aim of the current study*

The introduction includes technical terms that may not be accessible to all readers. Terms like "probability matrices," "reciprocal feedback," and "signal loss" are not adequately explained. While the audience is likely to be familiar with ecological and modelling terms, consider defining or briefly explaining more complex concepts, such as Bayesian belief networks and reciprocal feedback, to ensure clarity for all readers.

*We have revised these sections. The term ‘probability matrices’ has been removed and there are either definitions or ecological examples for the other terms used.*

Engage the reader by highlighting the practical implications of your work early on. For example, mention how the BBNet package could simplify the work of environmental researchers and practitioners, making complex modelling accessible to a broader audience.

*We have added sections which make this point throughout the introduction, in addition to this sentence, just before the study aim.*

As such, the ‘*BBNet*’ package puts tools for constructing and interpreting ecological models in the hands of a far broader number of environmental scientists and professionals than has previously been the case.

Theoretical basis: This section provides a comprehensive overview of Bayesian belief network (BBN) models and introduces the theoretical foundation of the BBNet package. To enhance clarity and structure, it is helpful to begin with a brief introductory sentence summarising the purpose of BBN models.

*We have modified the introduction to this section to read:* Bayesian belief networks (BBNs) are a modelling approach where interactions between different components of complex systems can be examined and predictions can be made for components of interest in these systems, as such, they can be used to make environmental or ecological predictions. *This is followed by an ecological example.*

Conclusion: The conclusion outlines the benefits and drawbacks of using BBN models in predictive ecological and environmental modeling. However, it would be enhanced by addressing the following points:
• The assertion that the approach is "rapid and easy to use and understand" for non-specialists, including stakeholders, may be overstated. Complex models, even those designed to be user-friendly, can pose significant challenges for those without a strong background in the relevant fields.

*We have modified this sentence to read:* We have presented an approach to predictive ecological and environmental modelling (which can link to social science outcomes) which can be rapid to develop, easy to use (albeit with some degree of training or troubleshooting support) and intuitive to understand key concepts and outcomes, particularly for non-specialists including stakeholders. *We have been using earlier forms of these models with undergraduate students for several years, including the published research demonstrating understanding of key processes by first year undergraduates in reference 22, so we hope this more cautious form of wording is now appropriate.*

• The discussion of potential future research directions or applications should be more detailed.

*We have tried to keep the conclusion short, as indicated in the instructions for authors’ section. However, we do point to an additional study which has used the BBNet package to address financial and political processes. While we envisage most readers of this manuscript will be environmentally focussed, and multiple examples of where this work has been used in this context are provided in the manuscript, we hope this additional work will illustrate some of the other uses and applications in other disciplines.*

• Ensure there is a logical flow from the summary of findings to implications and future directions.
• Consider using subheadings to organise different points for better readability.

*Response to both points above. As this is a relatively short section, we have not added subheadings, but have broken this up into paragraphs and slightly revised the structure, which now broadly follows:*

- *We have created a framework for simple and intuitive models (but see point above)*
- *Limitations of the models*
- *Advances of the ‘BBNet’ package*
- *Future use of the models*
