# Supplementary material for: Creating simple predictive models in ecology, conservation and environmental policy based on Bayesian belief networks
Source: PLoS One. 2024 Dec 10;19(12):e0305882. doi: 10.1371/journal.pone.0305882 (PMC11630608; doi:10.1371/journal.pone.0305882)
Supplement: Attachment — Submitted filename: Comments to the Author.docx [file pone.0305882.s007.docx]

**Response To Reviewer**

We thank the reviewer for their detailed comments on the manuscript. We are slightly confused as to why this paper was sent for a second round of review to a new reviewer, following us previously addressing another reviewer’s comments and providing a revision. We fully acknowledge the reviewer’s comments regarding the paper lacking a clear set of results, and reading more like an instruction manual. This was the purpose of the manuscript. As noted, previous studies (by some of the authors of this current paper) have used this modelling approach, and it has evolved and added new features over the last 9 years. To make to model as accessible as possible to the academic community, we have produced an R package, with similar predictive features to those used in previous studies, but with additional elements for network visualisation and understanding. The R package, along with this manuscript, and several training courses, should allow a wide uptake of the package for simple modelling tasks. We would argue that the basis of the predictive models has already been validated in previous literature, however the current paper presents an open access, user-friendly way to access this software, a protocol to follow for future studies, and the inclusion of the mathematical basis of the model also allows this manuscript to be a single citable source for the software. It is clear that there may be disagreement in the scope of the paper between the authors and reviewer, and we believe this can only be addressed by an editorial decision on the manuscript. However, we would also like to point out that the scope of the paper has not changed from the initial submission and editorial screening and that similar studies have been published within the journal within the last year – some examples below:

<https://journals.plos.org/plosone/article?id=10.1371/journal.pone.0297930>

<https://journals.plos.org/plosone/article?id=10.1371/journal.pone.0299993>

<https://journals.plos.org/plosone/article?id=10.1371/journal.pone.0309210>

We would therefore question why a manuscript this far along in the review process would therefore be considered unsuitable for the journal.

While we acknowledge the difference in scope of the article we have produced and what the reviewer has suggested, which does cover a number of the comments below, we also address each comment in turn (our response in italics).

Reviewer #2: General comment
As a kind of probabilistic graphical model BBNs became very popular to scientists and practitioners mainly due to the powerful probability theory involved, which makes them able to deal with a wide range of problems including environmental modelling. In this context the authors present a new modelling tool available as an R package specifically developed for environmental and ecological problems though claiming its applicability to other contexts.

Given the recognized technical complexity of BBNs outside the community of statistical modelers a theoretical introductory section would be beneficial for a valuable comprehension while for the theoretical basis of the presented model the authors redirect the reader to another scientific paper published in 2015 by one of the authors [8].

*We appreciate the line in question is poorly written and undermines the current study. As indicated in our overall aims, this paper* ***does*** *present the theoretical framework of the model, so it can be used as single point of reference for future studies. We have rephrased this to read:* The theoretical basis for the model is based on that in Stafford et al. (2015) [8] and is described below. A number of updates and additional useful tools are provided in the ‘*BBNet*’ package, described in the functions below (section 3), which provide additional functionality to understand and visualise the models and to examine information flow through the models.

According to the aim and scope of the Journal new software presentation are welcome provided that a proper validation is included demonstrating that the new tool achieves its intended purpose. This requirement may be met by including a proof-of-principle experiment or analysis when possible while the authors focused on presenting two datasets suitable for running the model and spent instead a few section intended as a user-manual likely more suitable for an appendix.

*We address this point above. However, the scope of the journal in terms of software articles states: “Submissions presenting methods, software, databases, or tools must demonstrate that the new tool achieves its intended purpose. If similar options already exist, the submitted manuscript must demonstrate that the new tool is an improvement over existing options in some way. This requirement may be met by including a proof-of-principle experiment or analysis; if this is not possible, a discussion of the possible applications and some preliminary analysis may be sufficient”. This is different the criteria stated by the reviewer, it is the improvement from a similar technique that may require a proof of principle experiment or analysis. As clearly stated, the aim of our paper is to: present (1) the underlying theory of the modified Bayesian belief networks, (2) introduce the ‘BBNet’ package as a user-friendly interface for ecological and environmental researchers and practitioners with limited modelling experience to produce useful and meaningful models, and (3) suggest a workflow for the formulation of these models, including parameterisation of the model and dealing with uncertainty. We believe the manuscript fulfils these goals.*

Despite the capabilities of the proposed R code including various functionality which are relevant for complex problem modelling (e.g. sensitivity analysis) the manuscript lack of any test case application thus making the overall presentation hard to be valued by a broader audience. Moreover the xls code presented in [8] seems to have similar predictive features so a comparison to highlight the improvements and/or proven advantage of the new R package over existing alternatives would be interesting too.

*The reviewer is right, the excel code in previous work has similar predictive abilities, as it is based on the same set of equations. Excel and R code has been used in many studies which have used this framework [8], [15],[16],[18], [20],[21],[29] in the revised reference list, which is not an exhaustive list. These provide context for the research in a wide range of ecological and environmental applications for those looking for test case examples. The purpose of this paper is to detail the new functionality in the R package, which was not present in these papers.*

Specific comments:
In section 2, Introduction, the BBNet is suddenly introduced here for the first time (except in the abstract) as if it was introduced somewhere before. It would be more readable to introduce it before talking about its features.

*The first line of the second paragraph of section 2 does mention the BBNet package when it could be more general, and we have rephased this. However, the manuscript does clearly introduce the BBNet package in the introduction, prior to specifics of the features being introduced in section 2.*

In section 2, Theoretical basis, it seems not usual to refer totally to a previous paper if this is relevant for the methodology adopted in this paper. This point was risen also in the general comment.

*See above – this was poorly phrased and has now been addressed.*

This section has to be thoroughly reviewed as the equations’ part follows the parameterization details making the reading hard and confusing.

*The equations do follow the details of what nodes and edges are, and the values they can take. This is essential information to give before the equations of the model. We do acknowledge that Table 1 does include some parameterisation information, and does come before the equations. However, table 1 also serves to illustrate the values the model can take. We would also argue that to achieve the main goals of the paper, most readers are unlikely to spend long looking at the mathematical equations, where as the details of the input parameters are essential for building and using the model, and should be placed first.*

Moreover concerning the mathematical formalism, the use of the i index for the increasing population may create confusion with the summation symbolism.

*We appreciate this point. However, while ‘i’ is common in summation, other values such as x and n are also frequently used. Using ‘i’ for increasing and ‘d’ for decreasing, and n for summation in our equations does seem logical, and we do clearly state:* “With subscripts i and d indicate increasing or decreasing respectively for the nodes.”

Section 3 reports details on the software availability, download repository and installation which are commonly reported after the conclusion section.

Bub-sections 3.1 and 3.2 are more written as a user manual than a science paper. They could be more fitted for an appendix than the body of the paper.

*As indicated above, we do appreciate this comment and acknowledge it, but this ‘user manual’ approach is our intention in the paper.*

Section 4 is dedicated to the model implementation with its functionalities, features and parameterization. Once again, the paper is hard to follow. Without graphical feature and tables, possibly related to some real case application and validation, the presentation of the BBN model capability may appear minimized.

*We agree that this could be made clearer. However, given the purpose of the paper, there is a tradeoff between being general (and therefore more useful as a longer-term resource) and being specific, and thus harder to generalise to readers’ own modelling needs. Figure 1 does show the Rocky Shore network model, and we now refer back to this in this section to clarify some of the points made. We also refer to supplementary material 3 again, which provides a run through of the model and discusses outcomes including graphical and numerical outputs. We hope this provides the necessary context for those who would require this.*

This is also evident in the Conclusion session where an intriguing sentence on the BBNet capability is hardly supported by the arguments developed in the manuscript. It is hard to justify the reasons for judging the value of this package without any case study with real data.

*We appreciate that the manuscript itself doesn’t provide evidence for all these claims. As such, we have cited literature where this modelling approach has been used in these contexts.*

Moreover, at the end of this section the authors are claiming the use of this model package in high level financial and political landscape of the UK referring to a paper that is not included in the reference list.

*This has been added. We appreciate this is a pre-print, it is currently under consideration for publication, also in PLoS ONE. Nevertheless it does illustrate the points of integrating multiple complex systems.*
